# Addressing the COVID-19 transmission in inner Brazil by a mathematical model

**DOI:** 10.1038/s41598-021-90118-5

**Published:** 2021-05-24

**Authors:** G. B. Almeida, T. N. Vilches, C. P. Ferreira, C. M. C. B. Fortaleza

**Affiliations:** 1 Medical School of Botucatu, São Paulo State University, Botucatu, 18618-687 Brazil; 2 Institute of Mathematics, Statistics, and Scientific Computing, University of Campinas, Campinas, 13083-859 Brazil; 3 Institute of Biosciences, São Paulo State University, Botucatu, 18618-689 Brazil

**Keywords:** Infectious diseases, Disease prevention, Health policy, Health services, Public health, Epidemiology

## Abstract

In 2020, the world experienced its very first pandemic of the globalized era. A novel coronavirus, SARS-CoV-2, is the causative agent of severe pneumonia and has rapidly spread through many nations, crashing health systems and leading a large number of people to death. In Brazil, the emergence of local epidemics in major metropolitan areas has always been a concern. In a vast and heterogeneous country, with regional disparities and climate diversity, several factors can modulate the dynamics of COVID-19. What should be the scenario for inner Brazil, and what can we do to control infection transmission in each of these locations? Here, a mathematical model is proposed to simulate disease transmission among individuals in several scenarios, differing by abiotic factors, social-economic factors, and effectiveness of mitigation strategies. The disease control relies on keeping all individuals’ social distancing and detecting, followed by isolating, infected ones. The model reinforces social distancing as the most efficient method to control disease transmission. Moreover, it also shows that improving the detection and isolation of infected individuals can loosen this mitigation strategy. Finally, the effectiveness of control may be different across the country, and understanding it can help set up public health strategies.

## Introduction

It has been a year since the first confirmed case of a novel coronavirus pneumonia in Wuhan, China. Now, the world experiences its very first pandemic of the globalized era. SARS-CoV-2 has rapidly spread through the currently connected continents, and the World Health Organization has declared a health emergency on international concern, which made many countries taking serious mitigation and suppression strategies^1^.

These strategies take importance when we look at the epidemic dynamics. The first studies estimated that the basic reproductive number of COVID-19 was 2.68 (95% CrI 2,47-2,86)^2^, which means one infected person can spread the virus to almost three people in a totally susceptible community. As there is no treatment or vaccine wide available, the best way to control the virus is to diminish social contact. China has shown to the world that when people stay at home, the virus circulation can be controlled, and we have more time for preparing health systems, producing individual protection equipment, developing research, and minimizing the consequences of the epidemic^3^. However, in Brazil, this kind of mitigation strategy (social distancing) does not work for self-employed people and low-income families since their maintenance depends on their own work. Besides, the number of people living in the same house can vary from 1.7 to 7.7 in the country. Only 52.5% of Brazilian households have basic sanitation and less than two residents per bedroom. Moreover, 6% of the Brazilian population lives in slums where access to safe water, basic sanitation, waste management, and hygienic conditions is not guaranteed^4–7^.

In Brazil, the introduction of COVID-19 happened later than in many other locations, and that gave us time to analyze all the new scientific evidence and the control measures taken overseas^8^. However, a country with continental dimensions cannot work with a single plan response. In the higher urban hierarchy cities, like São Paulo, for example, the disease spread initially from the medium and high levels of social classes to the lower ones^9^. However, what should be the scenario for inner Brazil, and what can we do on infection control in each of these locations?

Mathematical modeling has taken significant importance when applied to epidemics^10,11^. Since the earliest population studies on plague or measles, the methods have been refined. Today, with the parameters well established and more sensitive parameterization, such as contact patterns matrices, we may estimate how an epidemic will behave in a specific population and what should be our immediate response to the problem^12^.

Mathematical models may draw best and worst-case scenarios for a COVID-19 epidemic situation in small and medium cities in inner Brazil^13^. Our main objective is to study how the disease might behave in specific cities of the country and see what happens when combining two strategies: diminishing social contact plus testing and isolating positive cases. We point out that we are not aiming to characterize the temporal dynamics of the COVID-19 transmission in a city, state, or country, but highlight the difference in the disease transmission across the country and emphasize that control must be done differently in each one of these regions. Besides, we clustered the cities based on a set of characteristics in order to see if these were able to give us any clue about the disease dynamics. The consequences of relaxing restrictions are a theme of debate now, and in Brazil, it is happening before the epidemic’s peak has occurred, while the number of cases is still growing over the country. Mask wearing, mass testing, early detection of imported cases, and monitoring effective reproduction number are strategies that have been discussed and adopted around the world^14^.

## Results

**Figure 1:**
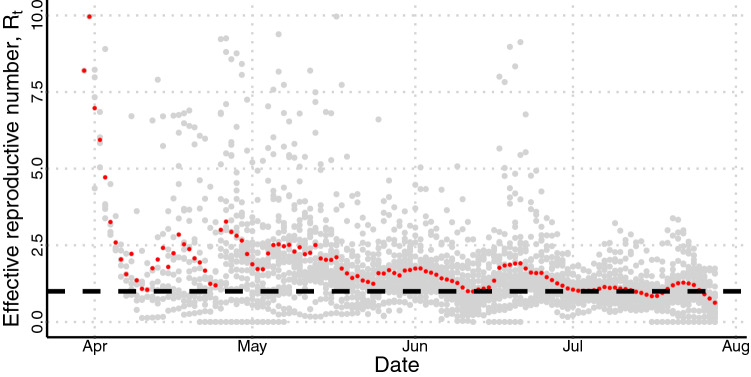
Temporal course of $R_t$ in each municipality involved in the study. In red, the average value, and in grey, the individual values. The dashed line shows the threshold of $R_t=1$. Above it, the transmission of the disease increases; below it, the disease’s transmission decreases.

The temporal evolution of the effective reproduction number $R_t$ is shown in Fig. 1. It was calculated for each municipality using data of daily incidence of cases and the knowledge about the generation interval of COVID-19^15^. In red, we plotted the average value, and in gray, the individual values. Mean $R_t$’s higher values were primarily observed at the beginning—absolute values from five to ten—which quickly changed to values between 1.8 and 2.7, after 10 days, since control measures were rapidly adopted across the country. It is crucial to notice that the large variability of $R_t$, and the oscillation, observed at each day when plotting cities together, can be associated with the fact that the cities started epidemic at different moments and the delay into report cases on weekends. Over time, $R_t$ slowly decreases until June. Although $R_t$ achieved values even lower than one during the epidemic’s course, this was not able to control the outbreak in any part of the country.

**Figure 2:**
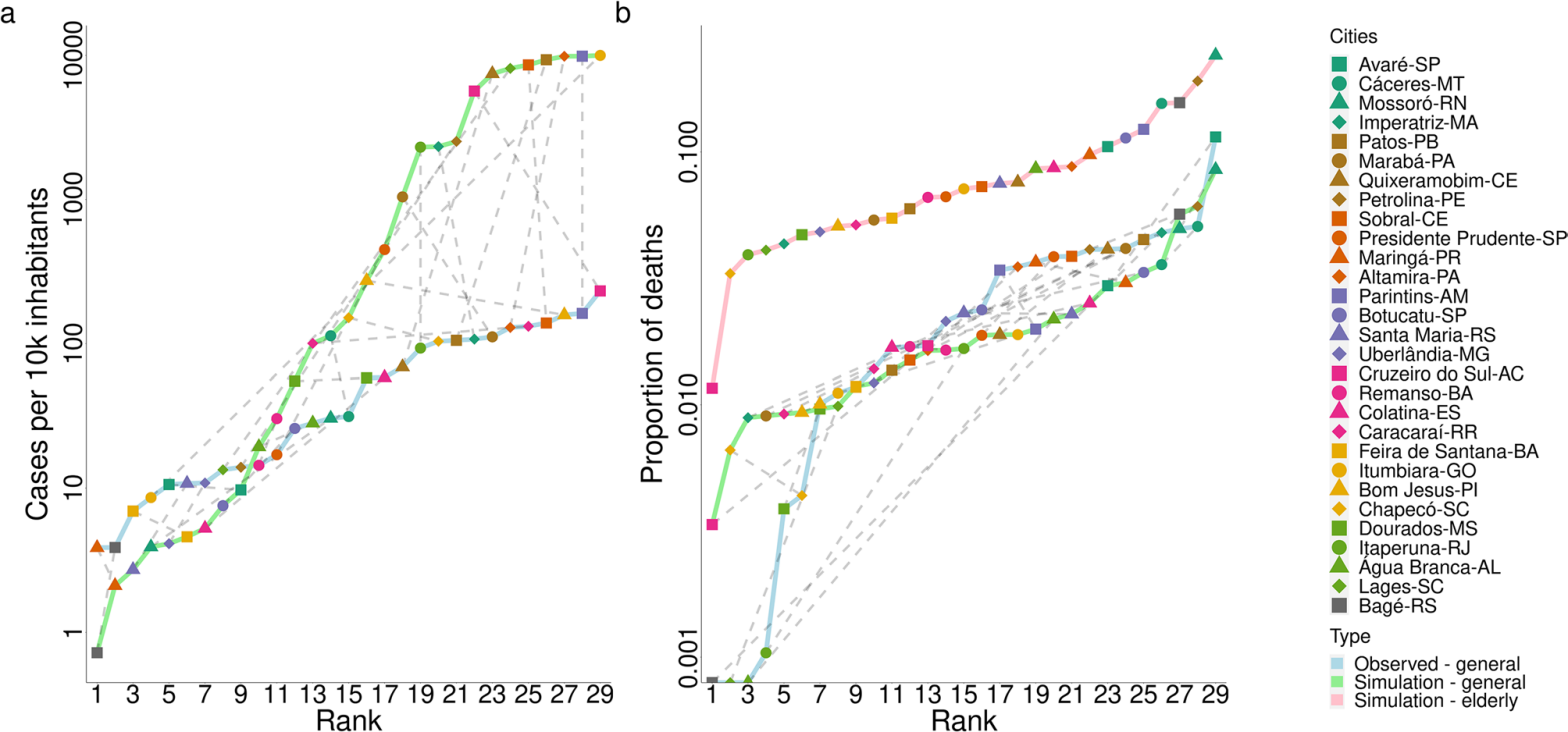
Simulation results and reported data for each municipality on the 60th day of the epidemic. In (**a**), we have the cumulative number of cases per 10,000 inhabitants versus city’s rank from the least infected to the most infected; in (**b**), the proportion of fatal cases versus city’s rank. The sum was done from day 1 to 60 of the epidemic course in each city. The first day was chosen to be the one at which the number of infected cases was higher than 10. The dotted grey lines connect the same city in the observed data and in the simulated data to highlight similarity on both results.

Figure 2 shows the municipalities ranked by the cumulative number of cases per 10,000 inhabitants, from the least to the most infected one until the 60th day of the epidemic, and also ranked by the proportion of fatal cases, i.e., the number of deaths divided by the number of cases. In Fig. 2a, we can see the cumulative cases for all infected individuals (the sum takes into account the number of infected individuals in all age class) obtained from the mathematical model (in green line), and from the reported data (in blue line). Following the dashed grey lines that connect both data, we can compare the simulations with laboratory-confirmed cases of COVID-19. Figure 2b shows the proportion of fatal cases obtained from reported data and from the mathematical model simulations. Pink lines focus measures obtained considering only individuals in age classes older than 50 years. Unfortunately, there is no available data regarding this population to be compared. The average distance between the cities’ observed ranks and their simulated ranks is $5.59\pm 6.87$ (median = 4) when comparing cases, and $7.93\pm 6.64$ (median = 6) when comparing deaths, both in all age classes.

**Figure 3:**
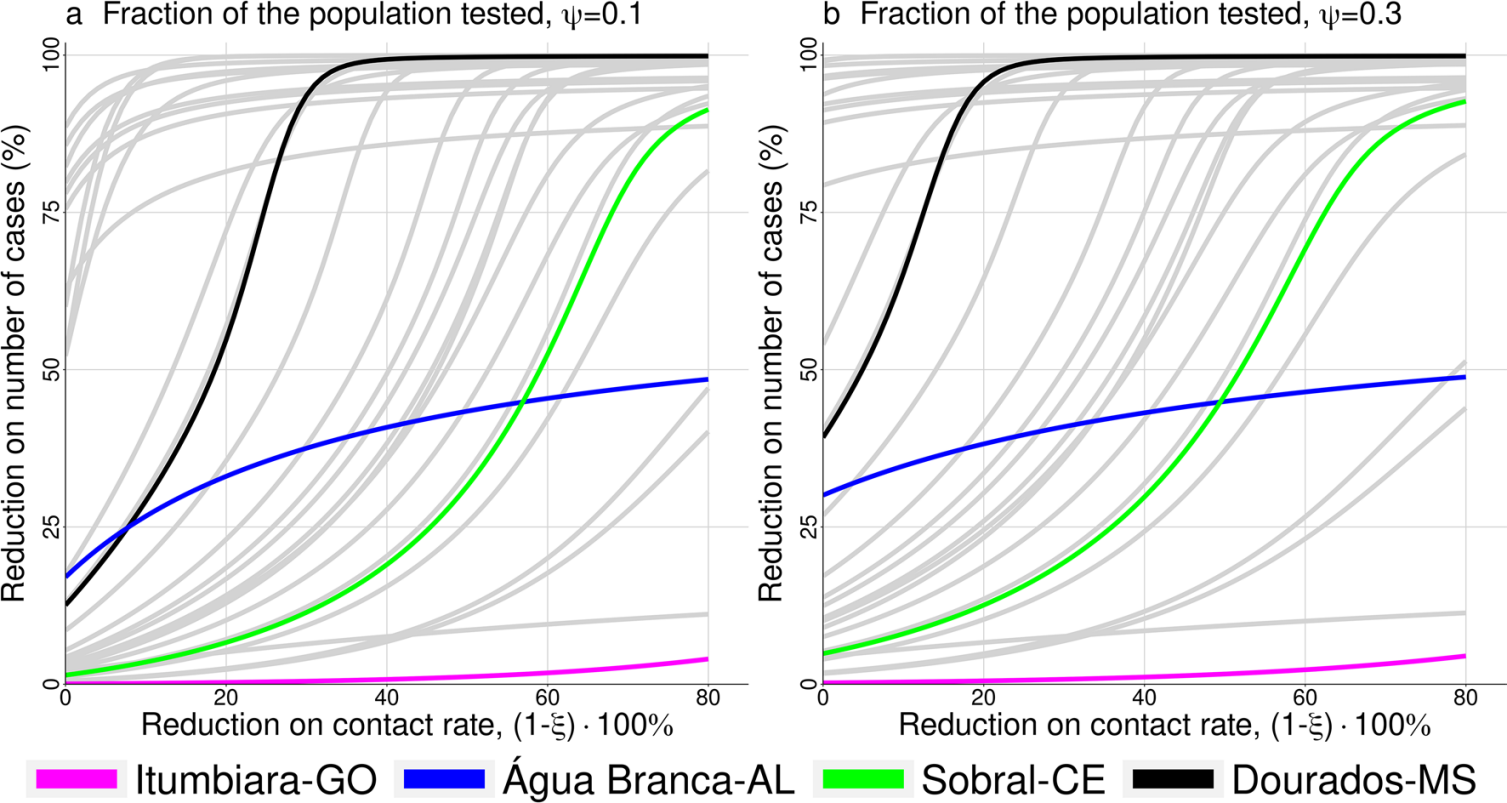
Reduction on the number of cases versus reduction on the contact rate, $1-\xi$, both in percentage. In (**a**), $\psi =0.1$ and in (**b**), $\psi =0.3$; where $\psi$ is the fraction of the population tested. Among the 29 municipalities involved in the study, we highlight four of them: Itumbiara, Água Branca, Sobral, and Dourados; the other ones are shown in grey lines.

Figure 3 shows the results when control measures are brought to the model, i.e., the percentage of reduction in the number of cases versus the reduction in contact rate, ($1-\xi$). The two panels were done for different values of $\psi$, where $\psi$ is the fraction of infected population tested, supposing late detection ($\tau =0.5$), $\nu =0.55$, and the other parameters are given in Table 2. In (a) we have $\psi = 0.1$ and in (b) $\psi = 0.3$. We highlighted four different cities based on their estimated basic reproductive number denoted, $R_0$ (Table 1); the other cities are displayed in grey lines. We can observe a large variation among control efficacy in the group of municipalities under study, reflecting the country’s heterogeneity, especially in inner Brazil. Considering only the four cities highlighted, we can see that reducing the contact rate by 20% cause a variation from 1 to 55% on reducing the number of cases. Moreover, this variation increases when the fraction of the population tested increases.

**Table 1:** Municipalities and key factors that may modulate COVID-19 transmission.

Municipality	Temperature	Humidity (%)	Density (inhab/km$^2$)	Population size inhabitants	$R_0$ (estimated)	HDI
Água Branca-AL	23.9	83.3	42.6	19,377	0.80	0.549
Altamira-PA	26.3	85.6	0.6	99,075	4.64	0.665
Avaré-SP	21.3	75.6	68.4	82,934	1.05	0.862
Bagé-RS	18.1	73.4	28.5	116,794	0.46	0.740
Bom Jesus-PI	26.2	61.9	4.1	22,629	1.50	0.668
Botucatu-SP	19.3	67.0	85.9	127,328	1.14	0.800
Cáceres-MT	19.3	75.0	85.9	87,942	1.69	0.708
Caracaraí-RR	27.2	80.1	0.4	18,398	1.22	0.624
Chapecó-SC	19.3	76.0	293.1	183,530	1.89	0.790
Colatina-ES	25.0	77.5	78.9	111,788	0.99	0.746
Cruzeiro do Sul-AC	25.7	84.5	8.9	78,507	2.71	0.510
Dourados-MS	22.3	77.6	48.0	196,035	1.67	0.747
Feira de Santana-BA	25.2	82.2	416.0	556,642	1.31	0.712
Imperatriz-MA	26.6	80.2	180.8	247,505	2.69	0.731
Itaperuna-RJ	23.3	76.6	86.7	95,841	2.53	0.730
Itumbiara-GO	24.3	72.7	37.7	92,883	6.65	0.752
Lages-SC	16.6	81.1	56.6	156,727	3.44	0.770
Marabá-PA	27.0	83.3	15.4	233,669	2.44	0.668
Maringá-PR	22.9	70.4	733.1	357,077	1.06	0.808
Mossoró-RN	27.7	81.4	123.8	259,815	1.16	0.720
Parintins-AM	27.0	86.3	123.8	102,033	4.67	0.658
Patos-PB	27.2	70.1	212.8	100,674	3.76	0.701
Petrolina-PE	25.4	60.1	64.4	293,962	2.95	0.702
Presidente Prudente-SP	24.0	66.3	368.9	207,610	2.25	0.806
Quixeramobim-CE	26.4	73.3	22.0	71,887	3.03	0.642
Remanso-BA	26.7	68.6	8.3	38,957	1.12	0.579
Santa Maria-RS	19.4	81.3	146.0	261,031	1.03	0.784
Sobral-CE	26.0	85.9	88.7	188,233	3.54	0.714
Uberlândia-MG	22.8	73.9	146.8	604,013	1.33	0.789

Each line brings the variables value of the city pointed in the first column. In the case of temperature and humidity the values are the average one observed in April month in each locality^31^. The other factors like density, population size, and Human Development Index (HDI) come from government’s website^13^; $R_0$ are estimated from data^32^.

**Figure 4:**
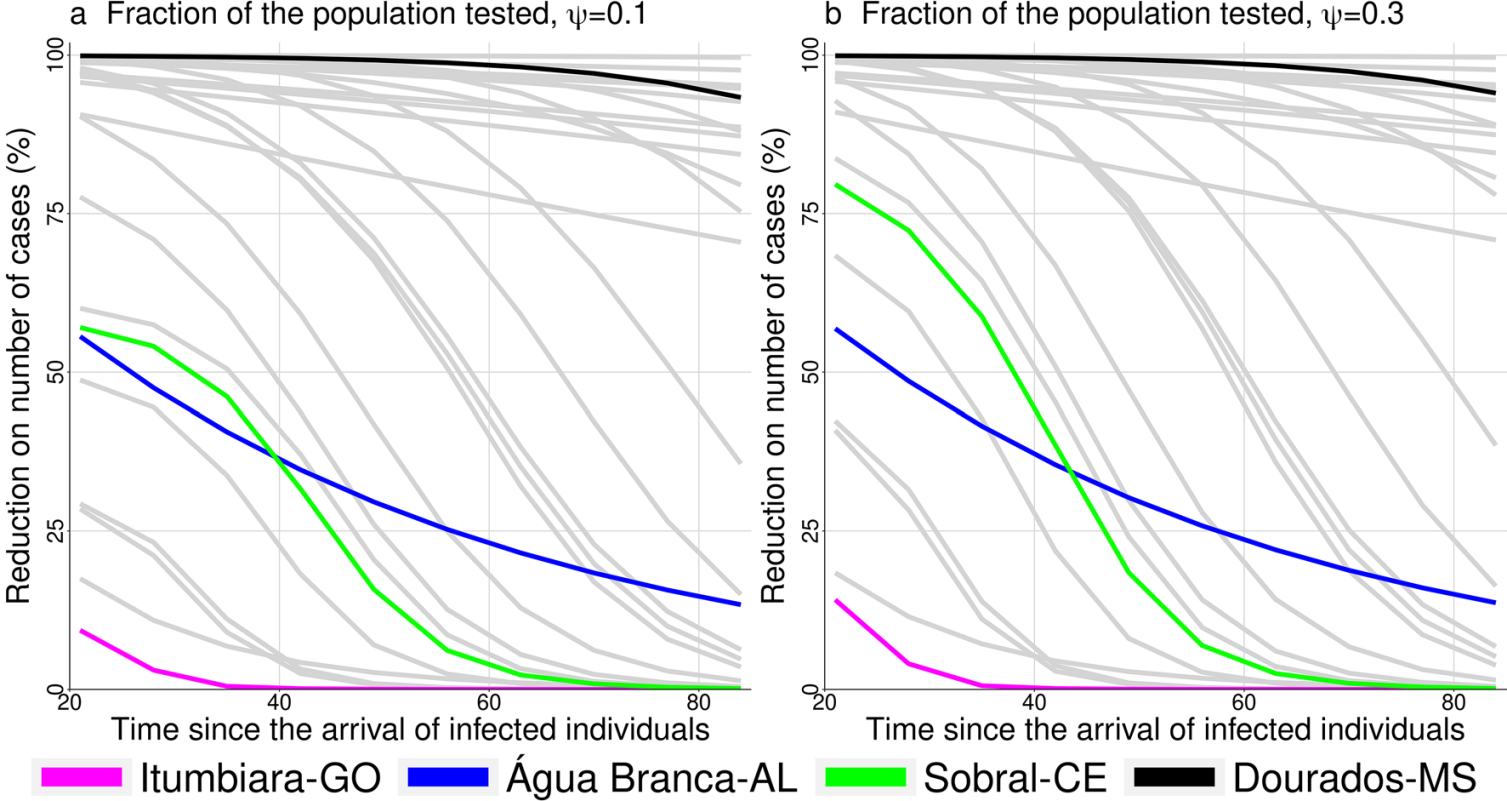
Reduction on the number of cases versus time of starting control. In (**a**), $\psi =0.1$ and in (**b**), $\psi =0.3$; where $\psi$ is the fraction of the population tested. Among the 29 municipalities involved in the study we highlight four of them: Itumbiara, Água Branca, Sobral, and Dourados; the other ones are shown in grey lines.

Figure 4 shows the reduction on the number of cases versus the time of starting control, $t_s$, in two scenarios that differs by the fraction of tested population, $\psi =0.1$ and $\psi =0.3$. The parameters are the same as in Fig. 3 with $(1-\xi )=0.6$, and the same cities are highlighted. The efficacy of control diminished as the time of control start is delayed. For some municipalities the reduction in the number of cases is less than 50%. In general, increasing the fraction of the population tested, control efficacy is increased.

**Figure 5:**
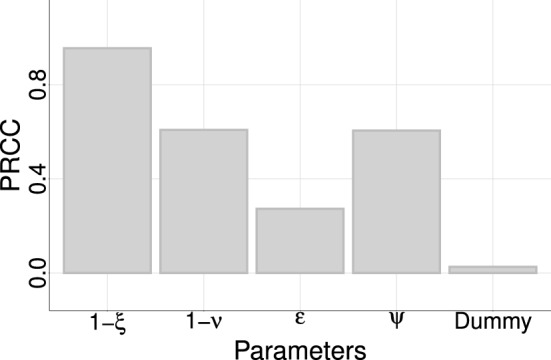
Sensitivity analysis using control efficacy as the output. A negative-control (dummy-parameter) was used to assign a zero value for a sensitivity index. Parameters values below the dummy are considered as not contributing to the model output. The result corresponds to the city of Sobral-CE, but the rank is obtained for the other cities.

The partial rank correlation coefficient (PRCC) obtained from a global sensitivity analysis^16^ is shown in Fig. 5. We run 3000 simulations that correspond to different input parameter sets, all of them related to control measures; the output is control efficacy. The parameters are $(1-\xi )$, $(1-\nu )$, $\epsilon$ and $\Psi$, respectively, the reduction of daily contacts among individuals, the reduction in the transmission rate of isolated individuals, the rate at which infected individuals are detected, and the fraction of infected individuals that are identified. As expected, the increase of any control measures promotes the increase of control efficacy, displaying positive values of correlation for any input parameter and the output. Nevertheless, the parameters contribute differently to it as can be seen by its absolute values, which rank them from less to more important (from lower to larger absolute value). We performed the analysis for all four highlighted cities in previous figures, and the results were the same. Here, we display the result for Sobral-CE.

**Figure 6:**
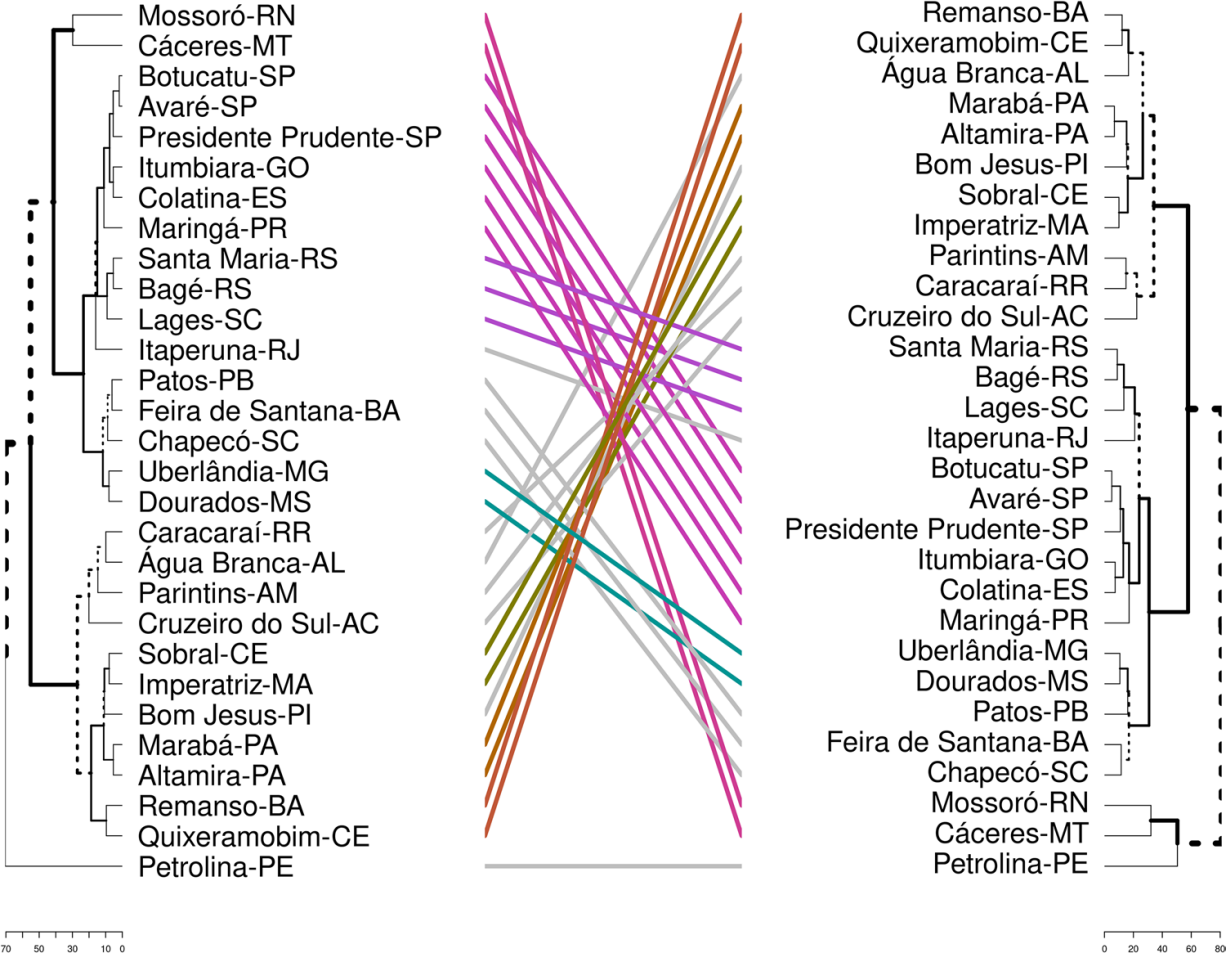
The municipalities are clustered in two ways, from left to right: (1) the proportion of fatal cases per age group and the age pyramid; (2) the same variables plus Human Development Index, population density, temperature, and humidity. The municipalities that changed group because of re-clustering are connected by gray line, while the ones that were kept together are connected through RGB color system.

Two different dendrograms were obtained from clustering the municipalities by their similarity, and they are shown in Fig. 6. The first one was built using the model’s input data, like the proportion of fatal cases per age group, and the age pyramid. In the second one, we included population density, human development index (HDI), as well as the value of temperature and humidity in April month. One city might belong to different clusters in each dendrogram, and the differences between the dendrograms are highlighted by gray lines connecting both. The distance among groups increased when we re-clustered them; in the new dendrogram, the groups are more dissimilar among them.

## Discussion

Our model shows epidemic dynamics for COVID-19 in various cities in inner Brazil. The epidemic’s dynamic features on each municipality were modeled by using local and regional epidemiological data, as the value of $R_0$, the proportion of fatal cases per age group, and sociodemographic data (age pyramid and social contact matrices). Mitigation strategies, such as social distancing of all individuals and detection followed by isolation of infected ones, were tested and compared. The cities were clustered, taking into account several variables that could influence disease transmission among individuals.

At the beginning of the epidemic, a substantial amount of the reported cases are imported cases. The data set does not distinguish between imported and local transmission cases, but the method used to evaluate $R_t$ takes into account the rising of infections coming from a local transmission already in course on the population. This can explain the high values seeing at the epidemic’s early stages (Fig. 1 and Table 1). Moreover, the available data displays the date of case report. Ideally, the calculation of the $R_t$ should be performed using the date of symptoms onset. Therefore, the limitation regarding the delay between symptoms onset and reporting must be considered. If we consider that the delay is somehow homogeneous across the country, the $R_t$ calculation is shifted in time. Besides mitigation strategies to halt or diminish disease transmission, deceleration in the initial epidemic’s growth rate can be driven by many factors like heterogeneity in population structure, behavior change of individuals, and increased herd immunity^17^.

Ranking the cities by the number of cumulative cases (Fig. 2a), we can see that, in general, the model provide a good prediction for disease behavior (as can be seen in Maringá, Altamira, and Cáceres), being the average distance between the rank of reported data and rank of simulated data of $5.59\pm 6.87$. It is important to highlight that those simulations were made considering no control measures. However, several cities in Brazil had enough time to implement social distancing and preventive measures after the arrival of the first case in São Paulo city, by February 2020. Adherence to social distancing, mask use, and self-isolation has been different across the country, but measure it is yet a challenge. Recently, several works have been trying to connect the transmission rate with mobility index, but a good model that link both measures is still missing^18^. This could explain the differences between the model’s prediction and the data collected. Among the cities that are in the top, six have a medium Human Development Index (HDI) and more than 85% of the population on the age classes until 50 years old. At least, regarding to simulations results, the rank follows with a good accuracy the one seen for the $R_0$ value. Further, as it was told, the available data refers to the date of case report. Once we are ranking the cities, if the delay in confirmation is homogeneous across the country, the results related to the cities’ rank must not change.

Regarding the proportion of fatal cases (Fig. 2b), since there is no available age-specific data for each city, our simulations use the reported lethality of COVID-19 at state level, which showed to be a good approach for most cities, such as Maringá and Chapecó. However, cities such as Bagé, Lages and Á gua Branca would have their mortality overestimated since they did not report any death until their 60th day of the epidemic. Here, the average distance between the rank of reported data and simulated data rank is $7.93\pm 6.64$. Interesting to note that among the cities that are in the top, five of them have very high HDI, and more than 16% of the population on the age classes older than 50 years old. In general, both results (Fig. 2) are in agreement with what is expected, medium HDI and youngest population explain the higher number of cases, while low HDI and older population are associated with higher fatality cases.

Overall, the fact that the model performs better for some localities compared to the other also reflects degrees of heterogeneity of COVID-19 test across the country^19^. Moreover, several local social and economic features can modulate the chance of death, not to forget access to health services and hospitals might be an important issue in each region of Brazil. Comparing the average distance between the rank of reported data (all age classes) with the rank of simulated data (only age classes older than 50 years old), we get $6.69\pm 6.29$ for fatal cases, which is a better result when compared to the previous one. We hypothesize that as the disease impacts the older age classes strongly, they are the responsible for most of the death and, therefore, the mortality rate might be consistent within the state.

It is expected that any kind of control on disease transmission will affect the epidemic’s course by delaying and reducing its peak. The gain on smaller numbers of infected individuals during the course of the epidemic is obtained by increasing its duration. Since there is no broad available vaccine, mitigation strategies rely on social distancing, isolation of infected individuals, self-isolation when you are a suspected case, mandatory quarantine applied to all populations, and travel restrictions^20,21^. So, we drew scenarios with different strategies and interventions. We can clearly see that we have an optimal control measure for each city, depending on the target. Hypothetically, let us consider that a reduction of 60% on the number of cases is needed to avoid the epidemic’s critical outcomes, such as health-system collapse. As we can see in Fig. 3, Itumbiara would not reach the needed reduction, probably because it has a high $R_0 ~(=6.65)$. In that case, it would be necessary to increase even more the control efforts. For Sobral and Dourados, both cities would reach the reduction on the number of infections, but with different control intensity, around 22% for Sobral and 63% for Dourados. This happens because Dourados has a lower $R_0$ than Sobral, 1.67 and 3.54 respectively. Água Branca is one particular case in which $R_0 < 1$, and this explains why control measures seem to be less useful. The variability of control efficacy is associated to country’s heterogeneity that may be quantified by its mean temperature (from 12$^{\circ }$ to 27$^{\circ }$ along the country), its population density (from 2.66 to 67.77 inhab/km$^2$), its human development index (from 0.450 to 1), and many other factors^22^. Eilersen and Sneppen discussed the cost-benefit of limited isolation and testing in COVID-19 mitigation^23^. Using an agent-based epidemiological model, they could compare several scenarios related to mitigation strategies such as testing and quarantining and concluded that this is much cheaper in terms of lost workdays than an extended lockdown. Also, the effect of quarantine on disease dynamics increases when testing is more widespread.

The effect of delaying the start of control measures was modeled as well (see Fig. 4). Again, the result shows a specific pattern at each municipality, but they all have in common one fact: the earlier cities start control, the greater is the reduction in the number of cases. Testing more people in the first 30 days is undoubtedly the best choice, and testing more people may also allow delaying social distance. Since the introduction of SARS-CoV-2 in Brazil, public laboratory certification for the molecular diagnosis of COVID-19 ranged from four laboratories to twenty-six in eight weeks. One can notice laboratories capacity is also increasing on time. This decrease the time of virus detection over the country, but in a heterogeneous way since there is a geographic concentration of laboratories in São Paulo state^19^. Amaku *et al*^24^ implemented a modified version of the classical SEIR compartmental model to compare two different test-trace-and-quarantine strategies to control the COVID-19 outbreak in the State of São Paulo, Brazil: indiscriminately testing the entire population of the State, and testing only symptomatic cases and their immediate contacts. They concluded that the second one is the most cost-effective strategy, and it can be applied especially in situations where social distancing is challenging to implement. Moreover, if the State of São Paulo had decided to adopt this strategy early, on April the 1st, it would have been possible to reduce the total number of cases by 90%.

The sensitivity analysis ranks the importance of parameters on control efficacy, which is (decreasing order): the reduction in the contact rate of the entire population due to control measures ($1-\xi$), the reduction in the contact rate of isolated individuals ($1-\nu$), the fraction of infected individuals that are identified ($\psi$) and the rate of testing ($\epsilon$), highlighting the importance of mandatory isolation and testing individuals for COVID-19 (see Fig. 5). Combining isolation of detected COVID-19 positive cases with social distancing can provide an efficient way of halting or diminishing disease incidence on population, but the control effectiveness will depend on each municipality’s characteristic. In Brazil (and other low-to-medium income countries), the expected peak of the disease was never observed; instead, it achieved a plateau sustained by a pattern of dispersion from major metropolitan areas to the interior^22^. Each state decides how to deal with a non controlled disease and an economy that may not support non-pharmacological control measures anymore. In São Paulo’s case, the territory is divided into seventeen health departments (DRS, in Portuguese) with respect to epidemiological control. Since the beginning of June, the state decided to adopt a reopening plan - that brings back people mobility and non-essential services - which can be more restrictive or more flexible, considering the growth rate of COVID-19 cases and deaths, and bed occupancy rates in each DRS. The same restrictive measures rule all cities belonging to a DRS, that can be adapted in response to the temporal-spatial behavior of the epidemic^25^.

We sustain the hypothesis that each city must be individually studied. However, it is possible to cluster cities (as it has been done in São Paulo state), considering similar characteristics, which ends up showing patterns of epidemic dynamics. In vast and heterogeneous countries like Brazil, we expect that many factors, such as population density, temperature, and mobility, modulate disease transmission. Quantifying and identifying such contributions can help governments to make decisions about mitigation strategies. The knowledge about other respiratory infection diseases that assault the population in different parts of Brazil, such as Influenza, can provide a pool of important information useful to forecasting COVID-19 in many municipalities.

Following this idea, in Fig.  6, the municipalities are clustered in two different ways. In the dendrogram on the left, we clustered cities by similar characteristics included in the model: the proportion of fatal cases by age group and age pyramid. Following the dendrogram we can identify three big groups: (I) Mossoró, Cárceres, Botucatu, Avaré, Presidente Prudente, Itumbiara, Colatina, Maringá, Santa Maria, Bagé, Lages, Itaperuna, Patos, Feira de Santana, Chapecó, Uberlândia, Dourados; (II) Caracaraí, Água Branca, Parintins, Cruzeiro do Sul, Sobral, Imperatriz, Bom Jesus, Marabá, Altamira, Remanso, Quixeramobim; (III) Petrolina. In each group, the average age and the average morality rate are, respectively, $32.7\pm 1.5$ and $0.019\pm 0.012$, $27.7\pm 1.8$ and $0.033\pm 0.015$, 28 and 0.030. Observe that groups II and III are very similar (when we compared them by the average values of age and mortality rate). The dendrogram on the right was generated including the cited characteristics plus new ones: temperature, humidity, population density, and HDI. This was done as an exercise to illustrate that we can add or remove characteristics from the clusters in order to find patterns, but it is essential to know which one of these characteristics is important on disease dynamic. For instance, comparing both dendrogram and Fig. 2, the re-clustering added Petrolina to the group of Cáceres and Mossoró, that display a similar number of cases and proportion of deaths; and Água Branca is set together with Quixeramobim and Remanso, being its number of cases between the number of cases of these two cities. Since the first clustering, Itumbiara, Água Branca, Sobral, and Dourados belong to different sub-groups and, therefore, have quite a different epidemic behavior. But, in the second clustering, the distance between Itumbiara and Dourados increases, while the distance between Água Branca and Sobral did not change too much, in accordance with Fig. 2. Moreover, Botucatu and Avaré belong to the same cluster and follow a similar epidemic evolution pattern. In summary, the two main groups that are identified can be distinguish by the HDI of their cities and average age of citizens. This emphasizes the statement that models that include, in some way, temperature, humidity, HDI and population density may better reflect the reality. This can spotlight groups of cities where it is expected that the control efficacy and the disease growth are similar. The results are sustained by Costa *et al.*^26^ that used a stochastic metapopulation model, inter-municipality mobility, and hypothetical mitigation scenarios, and showed that the diversity of outcomes related to the disease transmission in Brazil is observed in several geographical scales.

Like any other model, the approach developed here has its limitations. The fact that control measures may change the disease dynamic by decreasing or increasing its velocity of spreading may jeopardize model prediction. Also, spatial heterogeneity and social inequalities were not considered in the model, but it is known that in cities belonging to the higher urban hierarchy, COVID-19 spread first among the medium and high level of social classes, and afterward, it achieves the low social classes. Mitigation strategies, such as social distance and shelter-in, do not work for self-employed and low-incoming families, and to consider it would bring more complexity to the model. Moreover, the available data has two relevant limitations: testing is limited to symptomatic cases who seek health services, and the only date available in the data set is the date of the case report. No other relevant dates - i.e., exposure, onset, or laboratory confirmation - are available. These data limitations impact results diminished if we consider that the bias is homogeneous across the country. The bias generated by the under-reported data impacts our model’s parameter estimation, since the diagnosis capacity, compared to the number of cases in the population, changes over time. Recent works have demonstrated and have argued that the delay into case reports and the mitigation strategies may directly impact the $R_t$ estimation^27,28^. With more information about the available data and complex models, fitting the model parameters to the epidemic curve would be an interesting approach worthy of study.

However, here we were able to show that different control measures should be taken for different cities and, most importantly, each city may have an optimal combination of social distance with testing and isolating positive cases that control the epidemic’s curve and permit the health systems to be prepared for the peak of the number of cases. Cities in inner Brazil, such as Cruzeiro do Sul-AC, Imperatriz-MA, Altamira-PA, Bom Jesus-PI, and Parintins-AM that are clustered together, are susceptible to a delay in the arrival of the infections, and epidemic, which may decrease people’s risk perception and enhance the disease spreading^29^. As a consequence, those cities display a larger number of cases per number of inhabitants. We suggest the authorities to give special attention to those cities and perform an extensive educational campaign in order to control the infection. Our results also showed that testing and isolating people could perform a massive difference in controlling the epidemic. Due to a limited number of tests in Brazil, they have been mostly performed to confirm symptomatic cases, without a strategy of contact tracing. This plan should be revised, in accordance with other works^30^.

By a mathematical model and clustering cities, we suggest patterns of the evolution of the number of cases and control strategies for COVID-19 epidemic. As testing is a major issue for many nations at this moment of the pandemic, social distance in different degrees should be established.

## Methods

### Municipalities

**Figure 7:**
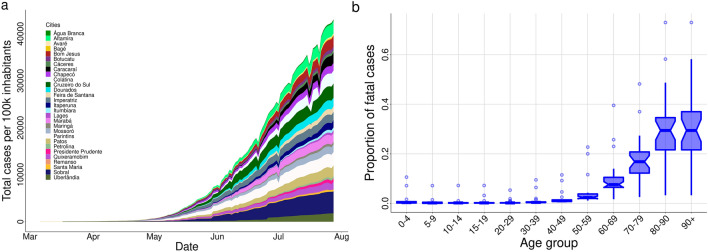
In (**a**), temporal evolution of the cumulative number of reported cases in each municipality; in (**b**), the boxplot of the proportion of reported fatal cases for different age groups in twenty Brazilian states enrolled in the study through their municipalities.

We aimed at a study capable of representing most small and medium cities of Brazil. Therefore, we decided to choose representative municipalities, with regional importance, from different states and regions, with varied population density, temperature, humidity, human development index (HDI), as well as age structure. From the North region we have: Altamira-PA, Marabá-PA, Cruzeiro do Sul-AC, Parintins-AM, Caracaraí-RR; from the Northeast region: Água Branca-AL, Sobral-CE, Quixeramobim-CE, Bom Jesus-PI, Imperatriz-MA, Mossoró-RN, Patos-PB, Petrolina-PE, Feira de Santana-BA, Remanso-BA; from the Central-West region: Dourados-MT, Cáceres-MT, Itumbiara-GO; from the South region: Santa Maria-RS, Bagé-RS, Lages-SC, Chapecó-SC, Maringá-PR; and from the Southeast region: Uberlândia-MG, Avaré-SP, Botucatu-SP, Colatina-ES, Itaperuna-RJ, Presidente Prudente-SP. Figure 7 shows each one’s geographic location on a Brazil map, with a heatmap showing the interpolation result (distance weighted interpolation) of the total number of cases per 100,000 inhabitants in those cities recorded on 28th July 2020. Table 1 summarizes some information about the cities listed in the present study. In particular, temperature and humidity correspond to the average values in April month^31^.

### Effective reproduction number

We calculated the effective reproduction number ($R_t$) for all chosen cities using the method proposed by Wallinga *et al.*^15^ and data of daily incidence of cases (*b*(*t*)), obtained from epidemic reports from each municipality^32^. We considered a simple susceptible-exposed-infected-recovered model with an average latent period ($\eta ^{-1}$) of 3.0 days and an infectious period ($\tau ^{-1}$) of 6.4 days, as well as the COVID-19’s mortality rate reported for each state ($\sigma$). The rates of leaving the exposed and infectious classes are denoted by $s_1 = \eta +\mu$ and $s_2 = \tau +\mu +\sigma$, where $\mu ^{-1}$ denote the life expectancy for Brazil. Therefore, the generation interval distribution (*g*(*t*)) is the combination of two exponential distributions $s_1e^{-s_1t}$ and $s_2e^{-s_2t}$ given by^33^

$$\begin{aligned} g(t) = \sum _{i=1}^2\frac{s_1s_2e^{s_it}}{\displaystyle {\prod _{j=1,j\ne 1}^2(s_j-s_i)}} \quad \text{ with } \quad t \ge 0. \end{aligned}$$

The duration of a generation interval is thereby implicitly specified as an exponential distribution with mean $T_c=1/s_1+1/s_2$. The expression above is valid when the infection force, $\Lambda$, satisfies the following inequality $\Lambda > \min (-s_1 , -s_2)$. Also, as we are dealing with a distribution, we need to normalize *g*(*t*). Using this equation we can evaluate $R_t$ as

$$\begin{aligned} R_t=\frac{b(t)}{\int _0^{\infty } b(t-a)g(a)da} \quad \text{ with } \quad \int _0^{\infty } g(t) dt = 1. \end{aligned}$$

The $R_0$ of each city was considered to be the average value of $R_t$ in the second week of the epidemic in the city. The first day was considered to be the one in which the cumulative incidence of infections reached ten cases. This choice was taken to guarantee that a local transmission was established in the city. We performed a spline interpolation and 7-day moving average on the data before used it to estimate $R_t$. The average value of $R_t$ at each calendar day from April to August can be seen in Fig. 1. It also displays the value obtained for each municipality (in grey points). Outliers were omitted from this plot.

### Clustering

After listing the cities, we clustered them in order to search for patterns. By taking each city as a model, studying the main characteristics, and crossing into a cluster study, we believe it is possible to extrapolate this study’s results to other cities that are not plotted here. We first grouped cities by their proportion of fatal cases per age group and age pyramid. Afterward, we added population density, temperature, humidity, HDI index and clustered them again. We used a hierarchical agglomerative clustering method, combining cluster threw the complete linkage criterion and Manhattan distance as a metric to measure dissimilarity between the observation sets^34^. The result is shown in Fig. 6.

### Data availability

Time series of the number of cases for each municipality in Brazil is not reported on any official government’s website. The Federal government does not provide it for open use. Therefore, we used daily cases reported on open sources in Brazil provided by a task force of volunteers (researchers and reporters) that compile the daily epidemiological reports of each state^32^. We used confirmed COVID-19 cases in the analysis, whose data refers to the date of case report and only mild and severe cases appear in this database (hospitalized cases and people seeking for medical assistance and health services).

**Figure 8:**
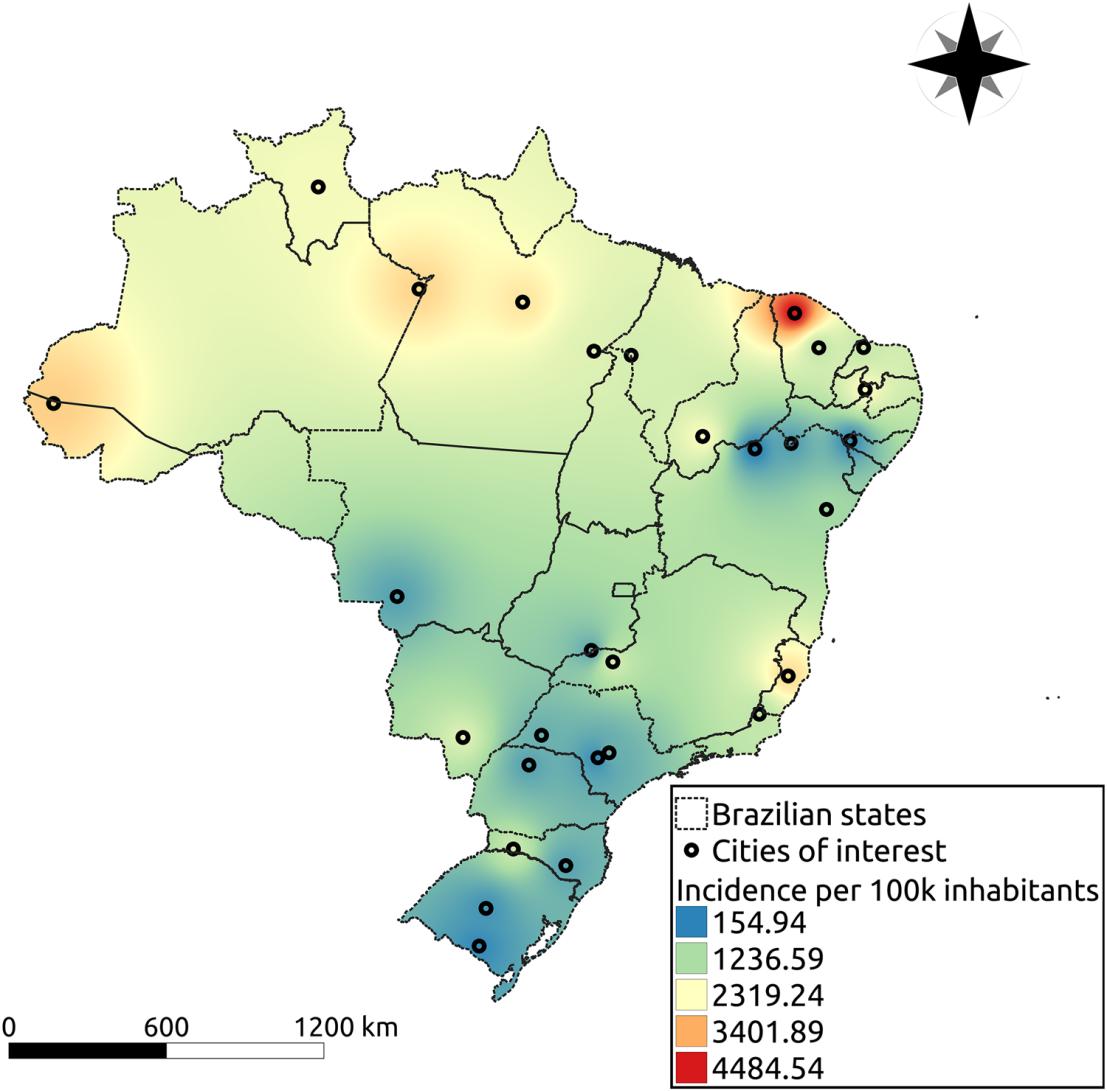
The geographic location of the municipalities enlisted in the study. The heatmap shows the interpolation result of the total number of cases per 100 thousand inhabitants in those cities recorded on July 28th. Cool colors mean less infected individuals while warm colors more infected individuals, and the scale goes from 153.6 (blue) to 4617.7 (red) cases per 100 thousand inhabitants. The cities were geocoded using the software Qgis (v3.10), and the interpolation was performed using the software’s tool for Inverse Distance Weighted Interpolation (https://www.qgis.org/en/site/).

Moreover, other issues may influence as well, such as the turnaround time of the performed tests and the fact that the data set does not distinguish between imported and autochthonous cases. To avoid the delay in reporting, we removed the last two weeks of data at the moment of the analysis. However, sub-notification is an issue that is difficult to be handle. Supposing that those issues occur in a homogeneous way throughout the country, we expected that the results would be impacted only by a scale factor, but keeping the conclusions regarding the temporal pattern of COVID-19 cases in each city. The age-dependent mortality is available separately in the epidemic’s reports from each state, but not for each city. In this work, we used data from 20 different states from Brazil to simulate 29 different cities. For each city, the reported time series of cases per 100 thousand inhabitants are shown in Fig. 8a, while the proportion of fatal cases in each age group is shown in Fig. 8b.

### Mathematical model

**Figure 9:**
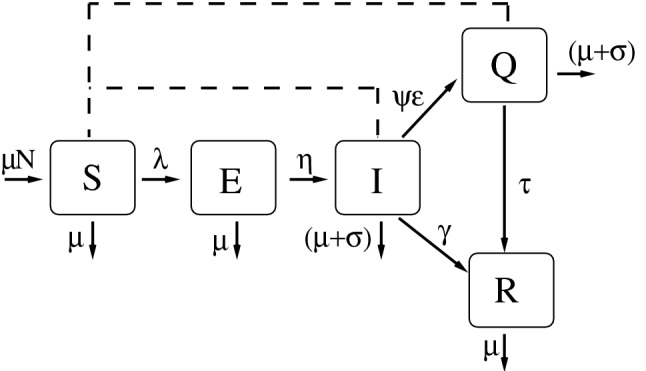
The variables of the model are susceptible (*S*), exposed (*E*), infected(*I*), isolated (*Q*) and recovered individuals (*R*). The continuous line indicates transitions between compartments and the dashed line indicates interactions between compartments that contributes to the infection force, $\lambda$. The model’s parameters are described at Table 2.

The proposed model is an age-structured one that divides the human population into fifteen age groups: 0 to 4 years, five years interval from 5 to 70 years, and greater than 70 years^35^. The variables of the model are $t, S_i:=S_i(t),E_i:=E_i(t),I_i:=I_i(t), Q_i:=Q_i(t), R_i:=R_i(t)$; respectively, time, susceptible, exposed, infected, detected and isolated infected individuals, and recovered one. The index *i* is the age class. The natural mortality rate $\mu$ appears in all age classes, and from 1 to 15, the parameter $\alpha _i$ takes into account the transition among them. Individuals are born susceptible, and they become exposed, when contacting infected or isolated individuals at rate $\beta _1$ and $\beta _2=\nu \beta _1$ ($\nu \in [0,1]$), respectively. The parameter $c_{i,j}$ represents the fraction of daily contacts that age group *i* has with age group *j*^36^. Target control can be done by varying $\xi _i\in [0,1]$, being $\xi _i=0$ complete protection of class *i* and $\xi _i=1$ no protection of class *i* against the infection. After a period of time $\eta ^{-1}$ exposed individuals becomes infectious. At rate $\epsilon$, a fraction $\psi \in [0,1]$ of infected individuals are identified and isolated. Additional mortality related to the disease is considered in the compartments of infected and isolated individuals, $\sigma _i$. Finally, these individuals become recovered at rates $\gamma$ and $\tau$. The ODE model is given by

1 $$\begin{aligned}&\frac{dS_{i+1}}{dt}=\mu N ~\delta _{i+1,1}+ \alpha _i S_i-\left( \beta _1\sum _{j=1}^{15} c_{i+1,j} ~\frac{I_j}{n_j}+\beta _2\sum _{j=1}^{15} c_{i+1,j} ~\frac{Q_j}{n_j}\right) \xi _{i+1} S_{i+1}-(\mu +\alpha _{i+1})S_{i+1} \nonumber \\&\frac{dE_{i+1}}{dt}=\alpha _i E_i+\left( \beta _1\sum _{j=1}^{15} c_{i+1,j} ~\frac{I_j}{n_j}+\beta _2\sum _{j=1}^{15} c_{i+1,j} ~\frac{Q_j}{n_j}\right) \xi _{i+1} S_{i+1}-(\mu +\alpha _{i+1}+\eta )E_{i+1} \nonumber \\&\frac{dI_{i+1}}{dt}=\alpha _i I_i+\eta E_{i+1} -(\sigma _i+\mu +\alpha _{i+1}+\gamma +\varepsilon \psi )I_{i+1} \\&\frac{dQ_{i+1}}{dt}=\alpha _i Q_i+\varepsilon \psi I_{i+1}-(\sigma _i+\mu +\alpha _{i+1}+\tau )Q_{i+1} \nonumber \\&\frac{dR_{i+1}}{dt}=\alpha _i R_i+\tau Q_{i+1}+ \gamma I_{i+1}-(\mu +\alpha _{i+1})R_{i+1} \nonumber \end{aligned}$$

with $i=0,...,14,~\alpha _0=\alpha _{15}=0$, $\alpha _1=...=\alpha _{14}=\alpha$, $\delta _{1,1}=1$, and $\delta _{j,1}=0$ with $j \ne 1$. Besides, $n_j=S_j+E_j+I_j+Q_j+R_j$, and $N=\sum _{i=1}^{15} n_j$ at $t=0$. Table 2 summarizes model parameters, their description, range of values and units^37,38^. Figure 9 shows the diagram of the compartmental model.

**Table 2:** Parameters of the model, their values (or range of values) and units^37,38^.

Parameter	Description	Value
$\mu$	Mortality rate	1/75 years$^{-1}$
$\sigma$	Additional mortality rate	[0.0, 0.20]
$\alpha$	Transition rate among age classes	1/5 years$^{-1}$
$\eta ^{-1}$	Latent period	3 days
$\gamma ^{-1}$	Infectious period	6.4 days
$\tau ^{-1}$	Isolation period	$\{1, 2, 5, 6\}$ days
$\epsilon$	Detection and isolation rate	1/3 days$^{-1}$
$\psi$	Fraction of infected that are detected	[0, 1]
$\xi , \nu$	Reduction on the infection transmission	[0, 1]
$\beta _1$	Transmission rate	[0.4397, 0.4782] days$^{-1}$
$\beta _2$	Transmission rate	[0.241835, 0.26301] days$^{-1}$

Defining $\bar{S}=S/N$ we can rewrite (1) as

2 $$\begin{aligned}&\frac{d\bar{S}_{i+1}}{dt}=\mu ~\delta _{i+1,1}+ \alpha _i \bar{S}_i-\left( \beta _1\sum _{j=1}^{15} c_{i+1,j} ~\frac{\bar{I}_j}{\bar{n}_j}+\beta _2\sum _{j=1}^{15} c_{i+1,j} ~\frac{\bar{Q}_j}{\bar{n}_j}\right) \xi _{i+1}\bar{S}_{i+1}-(\mu +\alpha _{i+1})\bar{S}_{i+1} \nonumber \\&\frac{d\bar{E}_{i+1}}{dt}=\alpha _i \bar{E}_i+\left( \beta _1\sum _{j=1}^{15} c_{i+1,j} ~\frac{\bar{I}_j}{\bar{n}_j}+\beta _2\sum _{j=1}^{15} c_{i+1,j} ~\frac{\bar{Q}_j}{\bar{n}_j}\right) \xi _{i+1} \bar{S}_{i+1}-(\mu +\alpha _{i+1}+\eta )\bar{E}_{i+1} \nonumber \\&\frac{d\bar{I}_{i+1}}{dt}=\alpha _i \bar{I}_i+\eta \bar{E}_{i+1} -(\sigma _i+\mu +\alpha _{i+1}+\gamma +\varepsilon \psi )\bar{I}_{i+1} \\&\frac{d\bar{Q}_{i+1}}{dt}=\alpha _i \bar{Q}_i+\varepsilon \psi \bar{I}_{i+1}-(\sigma _i+\mu +\alpha _{i+1}+\tau )\bar{Q}_{i+1} \nonumber \\&\frac{d\bar{R}_{i+1}}{dt}=\alpha _i \bar{R}_i+\tau \bar{Q}_{i+1}+ \gamma \bar{I}_{i+1}-(\mu +\alpha _{i+1})\bar{R}_{i+1} \nonumber \end{aligned}$$

with $\bar{n}_j=\bar{S}_j+\bar{E}_j+\bar{I}_j+\bar{Q}_j+\bar{R}_j$. The disease free equilibrium is given by

$$\begin{aligned} P_0=\left( S^*_1,0,0,0,0,...,S^*_{15},0,0,0,0\right) \end{aligned}$$

where

$$\begin{aligned} S^*_1=\frac{\mu }{\mu +\alpha _1},~S^*_i=\frac{\alpha _i}{\mu +\alpha _i}\quad \text{ with } ~ i\in {2,...,15}. \end{aligned}$$

In order to obtain the next generation matrix^39,40^, we used the reduced system given, in its vectorial form, by

3 $$\begin{aligned}&\frac{d\bar{\mathbf {E}}}{dt}= \mathscr {A} \bar{\mathbf {E}}+\text{ diag }(\beta _1\varvec{\xi })\text{ diag }(\bar{\mathbf {S}})~ \mathscr {C}~\text{ diag}^{-1}({\bar{\mathbf {n}}}) {\bar{\mathbf {I}}}+\text{ diag }(\beta _2\varvec{\xi })\text{ diag }(\bar{\mathbf {S}})~ \mathscr {C}~\text{ diag}^{-1}(\bar{\mathbf {n}}) \bar{\mathbf {Q}}-\text{ diag }(\varvec{\mu }+\varvec{\alpha }+\varvec{\eta })\bar{\mathbf {E}} \nonumber \\&\frac{d\bar{\mathbf {I}}}{dt}=\mathscr { A} \bar{\mathbf {I}}+\text{ diag }(\varvec{\eta }) \bar{\mathbf {E}}-\text{ diag }(\varvec{\sigma }+\varvec{\mu }+\varvec{\gamma }+\varvec{\varepsilon } \psi +\varvec{\alpha }) \bar{\mathbf {I}} \\&\frac{d \bar{\mathbf {Q}}}{dt}=\mathscr { A} \bar{\mathbf {Q}}+\text{ diag }(\varvec{\varepsilon }\psi ) {\bar{\mathbf {I}}}-\text{ diag }(\varvec{\sigma }+\varvec{\mu }+\varvec{\alpha }+\varvec{\tau })\bar{\mathbf {Q}}.\nonumber \end{aligned}$$

Bold symbols represent vectors as $$\varvec{x}=[x_1,...,x_{15}]^T$$ and diag$$(\varvec{x})$$ represent diagonal matrices, $$ M=[m_{ij}]$$, in which $$ m_{ii}=x_i$$. $$\mathcal {C}$$ is the contact-distribution matrix among the age groups^41^, and

$$\begin{aligned} \mathcal {\mathcal A}=\left( \begin{array}{cccccc} 0 &{}0 &{}0 &{}\hdots &{}0 &{}0\\ \alpha _1 &{}0 &{}0 &{}\hdots &{}0 &{}0\\ 0 &{}\alpha _2 &{}0 &{}\hdots &{}0 &{}0\\ 0 &{}0 &{}\alpha _3 &{}\ddots &{}0 &{}0\\ \vdots &{}\vdots &{}\ddots &{}\ddots &{}\vdots &{}\vdots \\ 0 &{}0 &{}0 &{}\hdots &{}\alpha _{14} &{}0 \end{array}\right) . \end{aligned}$$

The matrix of infection terms, $\mathcal F$, and the matrix of transition terms, $\mathcal V$, are given, respectively, by

$$\begin{aligned} \mathscr F=\left( \begin{array}{ccc} 0_{15\times 15} &{}\text{ diag }(\beta _1 \varvec{\xi })\text{ diag }(\bar{\mathbf {S}}^*)~\mathscr C~\text{ diag}^{-1}(\bar{\mathbf {n}}) &{}\text{ diag }(\beta _2\varvec{\xi }) \text{ diag }(\bar{\mathbf {S}}^*)~\mathscr C~\text{ diag}^{-1}(\bar{\mathbf {n}})\\ 0_{15\times 15} &{}0_{15\times 15} &{}0_{15\times 15} \\ 0_{15\times 15} &{}0_{15\times 15} &{}0_{15\times 15} \\ \end{array}\right) \end{aligned}$$

and

$$\begin{aligned} \mathscr V=\left( \begin{array}{ccc} \text{ diag }(\varvec{\mu }+\varvec{\alpha }+\varvec{\eta })-\mathscr {A} &{}0_{15\times 15} &{}0_{15\times 15}\\ -\text{ diag }(\varvec{\eta }) &{}\text{ diag }(\varvec{\sigma }+\varvec{\mu }+\varvec{\gamma }+\varvec{\alpha }+\varvec{\epsilon } \psi )-\mathscr {A} &{}0_{15\times 15}\\ 0_{15\times 15} &{}-\text{ diag }(\varvec{\epsilon }\psi ) &{}\text{ diag }(\varvec{\sigma }+\varvec{\mu }+\varvec{\alpha }+\varvec{\tau })-\mathscr {A} \end{array}\right) , \end{aligned}$$

in which $\bar{\mathbf {S}}^* =\mu \left[ \text{ diag }(\varvec{\mu }+\varvec{\alpha })-\mathscr {A}\right] ^{-1}\varvec{\delta }$, with $\varvec{\delta }=[1,0,...,0]^T$, is the disease-free equilibrium of (2). The basic reproductive number denoted by $R_0$ is given by the spectral radius of the next generator operator matrix given by $\mathscr {FV}^{-1}$ (i.e. its dominant eigenvalue). The disease-free equilibrium $\bar{\mathbf {S}}^*$ is locally asymptotically stable if $R_0<1$, and unstable if $R_0>1$. $R_0$ is the mean number of secondary cases that a primary case generates in a whole susceptible population, which implies before control measures. A simple and direct way to calculate the effort to be done to control an epidemic is given by $P_c=1-1/R_0$, where $P_c$ is the fraction of population that likely to be infected without mitigation. This represents the worst scenario since the deterministic approach has several assumptions like large population, well-mixed individuals, and no spatial structure.

### Simulations

In all simulations, the parameter $\beta _1$ was calibrated, for a given $R_0$ (Table 1), using the next-generation matrix, under no control measure. The addition mortality rates (days$^{-1}$) are calculated through the expression

$$\begin{aligned} \sigma _i=-\gamma \ln (1-p_i), \quad i=\{1,...,15\} \end{aligned}$$

where $p_i$ is the probability that an individual at age group *i* dies during their infectious period. For each city, we used data reported from their states to estimate $p_i$ (see Fig. 8b).

The simulations start with ten infected individuals (in the age class of 25 to 50 years) introduced in a wholly susceptible population. Control started later, after one month since the introduction of infected individuals. Control was explored by reducing contact rate among age classes (using the parameter $\xi$), decreasing the time of detection of infected individuals ($\varepsilon ^{-1}$), increasing the fraction of individuals that are detected and isolated ($\psi$), and decreasing the contribution of detected and isolated individuals to the disease transmission ($\nu$).

Two different scenarios were analyzed. The first one deals with a situation where the detection and isolation of infected individuals occur quickly. Therefore, we set up $\varepsilon ^{-1}$ to 1 and 2 days and $\tau ^{-1}=6 ~(\approx \gamma ^{-1})$ days. The second one suppose that detection takes longer time, then $\epsilon ^{-1} ~(\approx \gamma ^{-1})$ was set up to 5 and 6 days and $\tau ^{-1}=2$ days. The other parameters are $\beta _2=0.55\beta _1$ days$^{-1}$, $\eta ^{-1}=3$ days, $\gamma ^{-1}=6.4$ days, and $\mu =3.65\times 10^{-5}$ days. In general, figures were done with the set of parameters that represent the late detection.

Since the time of starting control impacts the evolution of disease transmission, the efficacy of control was measured varying this parameter in the simulation. For this, we measure the reduction (in percentage) on the number of infected individuals with and without control. Target and no target control over higher age classes was explored by ranking and comparing the municipalities by the cumulative number of infected individuals, and by the proportion of lethal cases. Finally, a sensitivity analysis based on partial rank correlation coefficient (PRCC) was done to discuss the contribution of each model control parameter to the control efficacy, measured as the percentage of infected cases that are avoided. The PRCC measures the monotonic relationship between an input parameter and the output variable when the linear effects of other independent variables are discounted^16^. The input parameters were $\epsilon , \xi , \nu ,$ and $\psi$; and they were sampled using the Latin Hypercube Sampling method. The first one took from a uniform distribution from 0.166 to 0.2 (late detection) and from 0.5 to 1 (early detection), and the others one from an uniform distribution in the range of 0 to 1. A PRCC close to one means that the input parameter and the output are strong and positively related, while negative values stand for negative correlation.
